# Lipoprotein(a) levels in Finnish adults: distribution and associations with other cardiovascular risk factors and their awareness and control

**DOI:** 10.1186/s12944-026-02938-x

**Published:** 2026-04-02

**Authors:** Alpo Vuorio, Anniina Ojanen, Tarja Palosaari, Tuija Jääskeläinen, Pekka Jousilahti, Maija Ruuth, Terhi Vihervaara, Mari Savolainen, Lara Lehtoranta, Petri T Kovanen, Annamari Lundqvist

**Affiliations:** 1 Department of Forensic Medicine, University of Helsinki, Helsinki, Finland; 2 Finnish Institute for Health and Welfare, Helsinki, Finland; 3 Novartis Finland Oy, Espoo, Finland; 4 Wihuri Research Institute, Helsinki, Finland

**Keywords:** Lipoprotein(a), cardiovascular disease, dyslipidemia, cardiometabolic risk factors, population-based study, prevention

## Abstract

**Background:**

Elevated lipoprotein(a) [Lp(a)] is an important genetic risk factor for cardiovascular diseases (CVDs). Because Lp(a)-lowering therapies are limited, prevention focuses on identifying individuals with elevated Lp(a) and optimizing other modifiable risk factors. We aimed to assess the distribution of Lp(a) levels in Finnish adults and examine its association with other CVD risk factors, as well as the awareness, treatment, and control of dyslipidemia.

**Methods:**

Data were derived from the Healthy Finland health examination survey conducted in 2023, comprising a nationally representative sample of 5,484 adults. Lp(a) levels were categorized using a cut-point at 125 nmol/L. Other CVD risk factors included were dyslipidemia, abnormal glucose metabolism, hypertension, and obesity. Analyses were weighted taking into account the sampling design and non-participation to provide nationally representative results.

**Results:**

Mean Lp(a) levels were 41.7 nmol/L (95% CI 39.0–44.3) in men (M) and 41.9 nmol/L (39.7–44.1) in women (W). Elevated Lp(a) was observed in 11.0% of men and 10.4% of women. Dyslipidemia was more prevalent among individuals with elevated Lp(a) (M: 88.1% vs. 78.4% *p* = 0.003, W: 79.2% vs. 73.2% *p* = 0.030) but this association reversed after correcting cholesterol for Lp(a). No associations were found between Lp(a) and other cardiometabolic risk factors. Individuals with elevated Lp(a) had slightly lower unawareness (M: 42.3% vs. 47.5%, *p* = 0.180, W: 38.8% vs.48.4%, *p* = 0.042) and better treatment (M: 38.1% vs. 31.7%, *p* = 0.010, W: 29.2% vs. 24.7%, *p* = 0.090) of dyslipidemia than those with lower levels while no association was found between Lp(a) and dyslipidemia control (M: 81.4% vs. 84.1%, *p* = 0.520, W: 74.6% vs. 73.0%, *p* = 0.740).

**Conclusions:**

Approximately one in ten Finnish adults had elevated Lp(a), a lower prevalence than in many other European populations but still affecting a substantial share of the population. Elevated Lp(a) was associated with higher prevalence of dyslipidemia prior to Lp(a) correction, but not with other CVD risk factors, and these individuals also showed slightly greater awareness and treatment of dyslipidemia. These findings emphasize the need for comprehensive management of modifiable CVD risk factors to reduce the overall burden of CVDs.

**Supplementary Information:**

The online version contains supplementary material available at 10.1186/s12944-026-02938-x.

## Background

Lipoprotein(a) [Lp(a)] is a genetically determined lipoprotein particle composed of low-density lipoprotein (LDL) and a unique glycoprotein, apolipoprotein(a) [1]. Lp(a) concentrations are primarily regulated by the *LPA* gene, with minimal influence from environmental factors such as diet and lifestyle. Elevated Lp(a) levels have been consistently linked to an increased risk of atherosclerotic cardiovascular diseases (CVDs), including coronary artery disease, ischemic stroke, and peripheral artery disease [2]. Despite the strong association between elevated Lp(a) levels and cardiovascular risk, Lp(a) measurement is not routinely included in standard clinical lipid panels in Finland, hence individuals are in general unaware of their Lp(a) levels. Due to the limited availability of medications specifically targeting Lp(a) reduction, the primary strategy for mitigating CVD risk in individuals with elevated Lp(a) involves recognizing the associated risk and managing modifiable cardiovascular risk factors [3].

The interactions between Lp(a) and other established cardiovascular risk factors, such as dyslipidemia, hypertension, and diabetes, are complex and not fully elucidated. Some studies suggest that elevated Lp(a) levels may synergistically increase cardiovascular risk when combined with these factors, yet further research is needed to define the nature of these interactions and to develop integrated models that predict cardiovascular outcomes more accurately [4, 5]. Additionally, the development of targeted therapies to lower Lp(a) remains an area of ongoing investigation [6, 7].

Lp(a) concentrations exhibit substantial inter-individual variability, largely due to polymorphisms in the LPA gene, which give rise to lifelong stable differences in Lp(a) levels between individuals. Lp(a) levels in the general population follow a skewed distribution, with a small proportion of individuals having very high concentrations, while most individuals fall within the lower to mid-range [8]. Elevated Lp(a) levels (≥ 125 nmol/L) [9], are found in approximately 20–30% of the general population, although prevalence rates vary by ethnicity [8]. In certain populations, such as those of African descent, Lp(a) levels tend to be higher, while individuals of East Asian descent typically have lower concentrations [1]. Gender differences have also been observed, with women generally having slightly higher Lp(a) levels, particularly after menopause. However, these differences are usually modest [1].

Given the substantial variability in Lp(a) levels and the limited understanding of their interaction with other cardiometabolic risk factors, population-based data are needed to better inform prevention strategies. To address this gap — and in light of the underrecognition of elevated Lp(a) in Finnish clinical practice due to lack of routine measurement and effective therapies — the present study characterizes Lp(a) distribution, its associations with key cardiometabolic risk factors, and dyslipidemia awareness, treatment, and control across the wide Lp(a) concentration range measured.

## Methods

The study was based on a nationally representative Healthy Finland Study, health examination survey, conducted in 2023 [10]. The sample of Healthy Finland Study included a total of 9 870 adults aged 20 or over of whom 58% (*n* = 5 749) participated in the health examination. This article includes data from 2 506 men and 2 978 women who were not pregnant and had complete data for the following variables: blood pressure (BP), total cholesterol (TC), low- and high density lipoproteins (LDL-C, HDL-C), triglycerides (TG), lipoprotein(a) [Lp(a)], glucose, glycated hemoglobin (HbA1c), height, weight, and waist circumference.

This study was performed in line with the principles of the Declaration of Helsinki. The Healthy Finland Study was approved by the HUS Regional Committee on Medical Research Ethics (HUS/900/2022) in September 2022. All participants signed an informed consent.

Information on sex, age and education was obtained from national registries. High education was defined as having at least the lowest level of tertiary education (a total 14 years or more of education from the start of comprehensive education).

Participants were asked to fast for at least 4 h before the blood sample collection. The samples were stored frozen at − 70 °C until analyzed. Laboratory measurements were performed at Finnish Institute for Health and Welfare (THL) Biomarkers Laboratory, which is accredited by the Finnish Accreditation Service (FINAS) and fulfils the requirements of the standard SFS-EN ISO/IEC 17025:2005. The scope of accreditation covers all analyses, except Lp(a). All measurements were performed on a clinical chemistry analyser (Architect c8000; Abbott Laboratories, Abbott Park, Illinois, USA). The quality of the results of the series of analysis were ascertained by using controls, which were used to determine interassay coefficients of variation (CVs). The course of the study comprising ten months in 2023–2024, the precision between series expressed as CV% ± SD, the accuracy of the methods (mean bias% ± SD) are as follows: 1.1% ±0.3, 0.1% ±0.7 for TC, 1.7%±0.2 and 1.6%±1.9 for HDL-C, 1.4%±0.3 and 2.2%±2.4 for TG, and 2.1%±0.7 and 5.5%±1.5 for glucose, 2.2%±1.0 for Lp(a), and 1.3%±0.1 for HbA1c. The bias indicates the difference between the laboratory’s own result and the target value of the quality assessment sample and describes the laboratory’s systematic error.

Lp(a) was analyzed in serum using immunoturbidimetric assay (Randox Laboratories, Crumlin, County Antrim, United Kingdom) calibrated in nmol/L (traceable to the WHO/IFCC reference material (IFCC SRM 2B)). The measured values of Lp(a) ranged from 5 to 486 nmol/L and the detection limit was 5 nmol/L. The values below the limit were set to 4 nmol/L for the statistical analyses (20.0% of the Lp(a) values). Lp(a) levels were categorized using a cut-point at 125 nmol/L according to earlier European recommendations widely used at the time of study design [9]. TC and TG were analyzed in serum using enzymatic methods (cholesterol oxidase 4-aminoantipyrine (CHOD PAP for TC and glycerol phosphate oxidase (GPO) for TG), and HDL-C using homogenous method for direct measurement. LDL-C was calculated using Friedewald formula.

Use of lipid-lowering medication (Anatomical Therapeutic Chemical (ATC) codes C10AA or C10AB) was based on purchase of medicines register (Finnish Social Insurance Institution KELA) and having treatment for dyslipidemia was defined if the person had purchased medication in one year time prior to the survey. Dyslipidemia was defined as having TC ≥ 5 mmol/L, LDL-C ≥ 3 mmol/L, HDL-C < 1.0 mmol/L (men) or < 1.2 mmol/l (women), TG > 1.7 mmol/L and/or use of lipid-lowering medication. We calculated Lp(a) corrected LDL-C and TC by extracting 30% of total Lp(a) mass from LDL-C and TC to obtain Lp(a) corrected values as suggested in the previous studies [11]. Dyslipidemia corrected for Lp(a) was defined similarly than dyslipidemia above using the Lp(a) corrected values for LDL-C and TC.

Glucose was measured from plasma using enzymatic hexokinase method and HbA1c was measured from whole blood by enzymatic methods. Use of diabetes medication (ATC codes A10) was based on purchase of medicines register (KELA) if the person had purchased medication in one year time prior to the survey. Abnormal glucose metabolism was defined as having HbA1c ≥ 48 mmol/mol, glucose ≥ 7.0 mmol/L, and/or use of diabetes medication.

BP was measured twice using an automated oscillometric device (WatchBP Office Vascular, Microlife^®^) [10], and the mean of two measurements was used in the analyses. Hypertension was defined as having systolic BP ≥ 140 mmHg, diastolic BP ≥ 90 mmHg and/or the self-reported current use of antihypertensive medication. For those whose self-reported antihypertensive medication information was missing (*n* = 960), ATC codes C02CA, C02DC, C03, C07, C08, and C09 from the purchase of medicines register (KELA) were used to determine the use of medication, if the person had purchased medication in one year time prior to the survey.

Anthropometric measurements, including weight, height, and waist circumference (WC), were measured during health examinations by trained research staff following international standard protocols [10]. Obesity was defined as body mass index (BMI) ≥ 30 kg/m² and abdominal obesity as waist circumference ≥ 102 cm in men and ≥ 88 cm in women, both according to WHO classification [12].

Accumulation of risk factors was estimated by summing the following cardiometabolic risk factors: dyslipidemia, abnormal glucose metabolism, hypertension, and abdominal obesity. WC was used as the measure of obesity, as accumulating evidence [13, 14] indicates that abdominal obesity is a stronger predictor of cardiometabolic and cardiovascular risk than BMI.

Information on awareness of dyslipidemia was collected via a questionnaire. Of those 5 484 individuals included in the present article’s data, 82% had completed the questionnaire and were therefore eligible for the assessments of awareness of dyslipidemia. Unawareness of dyslipidemia was defined as having dyslipidemia and responding “no” or “I don’t know” to the question regarding a prior physician diagnosis or indicating that they had never had their cholesterol measured, i.e. lacking dyslipidemia diagnosis. Treatment of dyslipidemia was defined as the use of lipid-lowering medication (see above) among individuals with dyslipidemia, and control of dyslipidemia was defined as achievement of LDL-C < 2.6 mmol/L among individuals using lipid-lowering medication.

### Statistical analyses

All statistical analyses were conducted with R Statistical Software version 4.5.2 [15] using the survey package [16] and Stata version 16 (14, for age-adjusted estimates) taking into account the sampling design, sampling probabilities and non-response by using inverse probability weighting [17] to provide nationally representative results. Separate population weights were calculated for those that participated only the health examination and for those that responded also the questionnaire [10]. Age-adjusted and weighted means and prevalences and their 95% confidence intervals were obtained as model adjusted estimates based on the predictive margins [18] using linear or logistic regression models including the age as a categorical covariate (20–39, 40–49, 50–59, 60–69 and 70 + years) and stratified by sex. Odds ratios (ORs) were analyzed using logistic regression model with age as a continuous covariate and stratified by sex. We conducted analyses including also education as a covariate in the models. As this adjustment did not materially change the results, the results from age-adjusted models have been presented. The differences between the groups were tested using the adjusted Wald test.

## Results

**Fig. 1:**
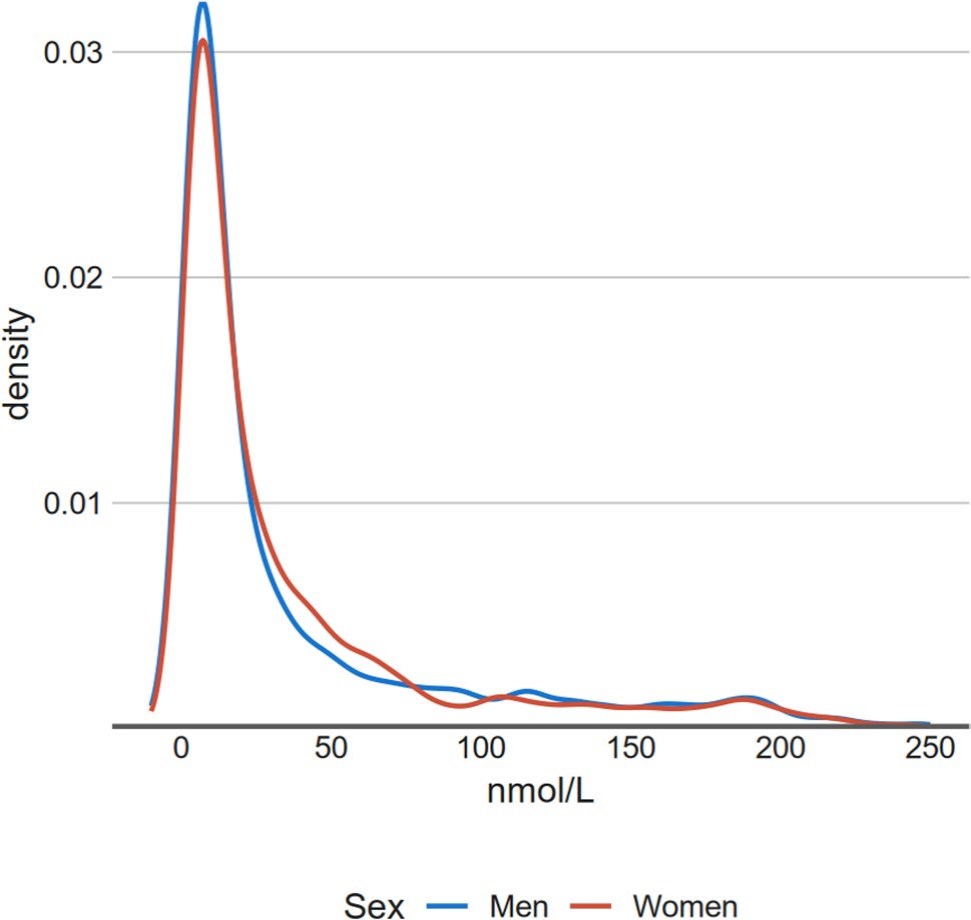
Distribution of Lp(a) concentrations in nmol/L among men and women* *X-axis is truncated to 250 nmol/L (*n* = 63 above that limit)

A total of 5 484 participants (46% men) with a mean age of 55 years were included. Baseline characteristics of the study population are shown in Table 1. Age-adjusted mean Lp(a) levels were 41.7 nmol/L (95% CI 39.0–44.3) in men and 41.9 nmol/L (95% CI 39.7–44.1) in women, with 11.0% of men and 10.4% of women exceeding Lp(a) level of 125 nmol/L. Distributions of Lp(a) concentrations by sex are presented in Fig. 1.

**Table 1:** Baseline characteristics of the study population by sex*

	Men (*n* = 2 506)	Women (*n* = 2 978)
Age, mean (SE)	52.8 (0.36)	53.0 (0.35)
High education,%	31.0	40.0
Lp(a) nmol/L, mean (SE)	41.7 (1.36)	41.9 (1.12)
Lp(a) ≥ 125 nmol/L, %	11.0	10.4
TC mmol/L, mean (SE)	4.9 (0.03)	5.1 (0.02)
TC mmol/L corrected for Lp(a), mean (SE)	4.8 (0.03)	4.9 (0.02)
HDL-C mmol/L, mean (SE)	1.3 (0.01)	1.6 (0.01)
LDL-C mmol/L, mean (SE)	2.9 (0.02)	2.9 (0.02)
LDL-C mmol/L corrected for Lp(a), mean (SE)	2.7 (0.02)	2.7 (0.02)
Triglyceride mmol/L, mean (SE)	1.6 (0.02)	1.3 (0.01)
Dyslipidemia, %^1^	79.5	73.7
Dyslipidemia corrected for Lp(a), %^2^	75.4	69.3
Systolic BP mmHg, mean (SE)	134.6 (0.44)	130.8 (0.41)
Diastolic BP mmHg, mean (SE)	80.9 (0.25)	78.0 (0.21)
Hypertensive medication, %	29.9	28.4
Hypertension, %^3^	52.2	44.0
Glucose mmol/L, mean (SE)	6.0 (0.03)	5.7 (0.02)
HbA1c mmol/mol, mean (SE)	37.1 (0.17)	36.6 (0.12)
Abnormal glucose metabolism, %^4^	13.5	9.6
BMI kg/m^2^, mean (SE)	27.8 (0.11)	27.6 (0.11)
Obesity, %	26.4	29.2
Waist circumference cm, mean (SE)	99.6 (0.30)	90.6(0.25)
Abdominal obesity, %	38.9	52.7
At least one risk factor, %^5^	87.4	83.1
At least two risk factors, %^5^	59.4	59.1
3–4 risk factors, %^5^	30.3	31.6

*age-adjusted means with standard errors (SE), and prevalences, %

^1^ TC ≥ 5 mmol/L and/or LDL-C ≥ 3 mmol/L and/or HDL-C < 1.0/1.2 mmol/L (men/women) and/or triglycerides > 1.7 mmol/L and/or lipid-lowering medication

^2^ Similar as ^1^ with TC and LDL-C corrected for Lp(a)

^3^ Systolic BP ≥ 140 mmHg and/or diastolic BP ≥ 90 mmHg and/or antihypertensive medication

^4^ HbA1c ≥ 48 mmol/mol and/or glucose ≥ 7.0 mmol/L and/or diabetes medication

^5^ Risk factors: dyslipidemia^1^, hypertension^3^, abnormal glucose metabolism^4^, and abdominal obesity (≥ 102/88 cm men/women)

Dyslipidemia was more prevalent among individuals with elevated Lp(a) compared with those with lower Lp(a), both in men (88.1% vs. 78.4%, *p* = 0.003) and women (79.2% vs. 73.2% *p* = 0.030, Table 2). The OR for dyslipidemia was 2.05 (95% CI 1.25–3.35) in men and 1.51 (95% CI 1.05–2.17) in women. However, after correcting dyslipidemia for Lp(a), the association in men attenuated and was no longer statistically significant (OR 0.79, 95% CI 0.56–1.13). Conversely, in women Lp(a) corrected dyslipidemia was less common in those with elevated Lp(a) than in those with lower levels (OR 0.66, 95% CI 0.49–0.91). Among men with elevated Lp(a) levels, 27.4% had elevated LDL-C corrected for Lp(a), while 39.5% in those with lower Lp(a) levels (*p* = 0.001). The corresponding proportions among women were 23.3% and 37.3% (*p* < 0.001).

**Table 2:** Prevalence of individual cardiometabolic risk factors and age-adjusted odds ratios by Lp(a) category*

Risk factor	Lp(a) < 125 nmol/L, % (95% CI)	Lp(a) ≥ 125 nmol/L, % (95% CI)	*p*-value	OR (95% CI)
Men				
Dyslipidemia^1^	78.4 (76.5, 80.4)	88.1 (83.2, 93.0)	**0.003**	**2.05** (1.25, 3.35)
Dyslipidemia corrected for Lp(a)^2^	75.7 (73.9, 77.6)	72.1 (65.8, 78.5)	0.259	0.79 (0.56, 1.13)
Abnormal glucose metabolism^3^	13.8 (12.1, 15.5)	11.7 (8.4, 14.9)	0.298	0.80 (0.54, 1.20)
Hypertension^4^	52.5 (50.0, 55.1)	49.4 (44.4, 54.4)	0.257	0.85 (0.64, 1.12)
Obesity^5^	26.7 (24.9, 28.6)	23.5 (18.5, 28.4)	0.259	0.82 (0.60, 1.11)
Abdominal obesity^6^	39.3 (37.1, 41.5)	35.7 (30.7, 40.8)	0.234	0.82 (0.63, 1.07)
Women				
Dyslipidemia^1^	73.2 (71.4, 74.9)	79.2 (74.2, 84.1)	**0.030**	**1.51** (1.05, 2.17)
Dyslipidemia corrected for Lp(a)^2^	70.0 (68.3, 71.7)	62.6 (57.5, 67.8)	**0.007**	**0.66** (0.49, 0.91)
Abnormal glucose metabolism^3^	9.7 (8.4, 10.9)	9.2 (6.3, 12.2)	0.795	0.91 (0.60, 1.41)
Hypertension^4^	43.6 (41.7, 45.5)	47.4 (42.8, 52.1)	0.104	1.26 (0.95, 1.68)
Obesity^5^	29.0 (27.4, 30.5)	30.8 (25.6, 36.0)	0.494	1.11 (0.85, 1.45)
Abdominal obesity^6^	52.7 (51.0, 54.5)	52.4 (46.5, 58.2)	0.915	1.00 (0.75, 1.33)

* *CI* confidence interval. *P* value from the adjusted Wald test for the difference in the prevalence of the Lp(a) categories. *OR* odds ratio using Lp(a) < 125 nmol/L as a reference group

^1^ TC ≥ 5 mmol/L and/or LDL-C ≥ 3 mmol/L and/or HDL-C < 1.0/1.2 mmol/L (men/women) and/or triglycerides > 1.7 mmol/L and/or lipid-lowering medication

^2^ Similar as ^1^ with TC and LDL-C corrected for Lp(a)

^3^ HbA1c ≥ 48 mmol/mol and/or glucose ≥ 7.0 mmol/L and/or diabetes medication

^4^ Systolic BP ≥ 140 mmHg and/or diastolic BP ≥ 90 mmHg and/or antihypertensive medication

^5^ BMI ≥ 30 kg/m^2^

^6^ Waist circumference ≥ 102 cm for men and ≥ 88 cm for women

No association was observed between Lp(a) levels and other cardiometabolic risk factors in either sex. Among men, the age-adjusted ORs were 0.80 (95% CI 0.54–1.20) for abnormal glucose metabolism, 0.85 (95% CI 0.64–1.12) for hypertension, 0.82 (95% CI 0.60–1.11) for obesity, and 0.82 (95% CI 0.63–1.07) for abdominal obesity. The corresponding ORs among women were 0.91 (95% CI 0.60–1.41) for abnormal glucose metabolism, 1.26 (95% CI 0.95–1.68) for hypertension, 1.11 (95% CI 0.85–1.45) for obesity, and 1.00 (95% CI 0.75–1.33) for abdominal obesity.

Among men, the accumulation of cardiometabolic risk factors was less common among individuals with elevated Lp(a) levels compared with those with lower levels (Table 3). The age-adjusted ORs of having 3–4 additional cardiometabolic risk factors were 0.72 (95% CI 0.56–0.94) among men with elevated Lp(a) levels. Among women, no such association was observed (OR 1.07, 95% CI 0.76–1.52).

**Table 3:** Prevalence of accumulation of cardiometabolic risk factors* and age-adjusted odds ratios by Lp(a) category**

Number of risk factors	Lp(a) < 125 nmol/L, % (95% CI)	Lp(a) ≥ 125 nmol/L, % (95% CI)	*p*-value	OR (95% CI)
Men				
At least one	86.9 (85.2, 88.7)	91.5 (87.1, 95.9)	0.106	1.69 (0.92, 3.09)
At least two	59.1 (56.6, 61.6)	61.7 (56.2, 67.1)	0.388	1.13 (0.84, 1.54)
3–4	30.9 (28.7, 33.0)	25.7 (21.7, 29.8)	0.031	**0.72** (0.56, 0.94)
Women				
At least one	82.8 (81.3, 84.3)	86.6 (82.3, 90.9)	0.123	1.47 (0.92, 2.35)
At least two	58.7 (57.0, 60.5)	62.6 (57.2, 67.9)	0.178	1.26 (0.90, 1.76)
3–4	31.5 (29.9, 33.1)	32.6 (27.5, 37.8)	0.654	1.07 (0.76, 1.52)

* Sum of the following risk factors: dyslipidemia, abnormal glucose metabolism, hypertension and abdominal obesity (see Table 2 for definitions)

** *CI* confidence interval. *P* value from the adjusted Wald test for the difference in the prevalence of the Lp(a) categories. *OR* odds ratio using Lp(a) < 125 nmol/L as a reference group

**Fig. 2:**
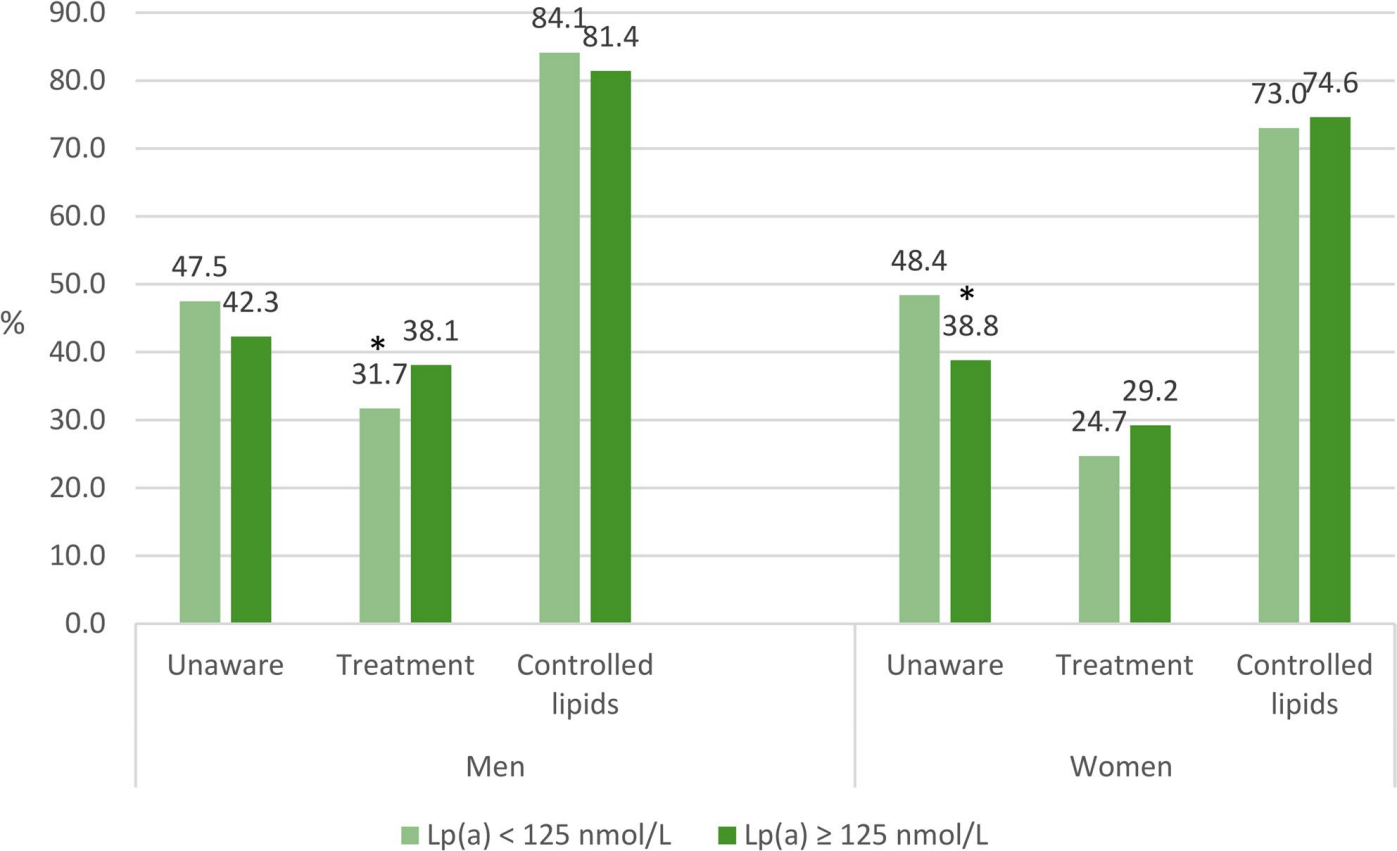
Prevalence of unawareness^1^, treatment^2^ and control^3^ of dyslipidemia among men and women by Lp(a) category^4^ ^1^Lacking diagnosis of dyslipidemia among individuals with dyslipidemia (information available for 1588 men and 1948 women with dyslipidemia) ^2^Lipid-lowering medication among individuals with dyslipidemia (information available for 2031 men and 2274 women with dyslipidemia) ^3^LDL-C < 2.6 mmol/L among individuals with lipid-lowering medication (information available for 681 men and 653 women with lipid-lowering medication) ^4^Analyses were restricted to individuals with dyslipidemia. * *p* value < 0.05 for the difference of the Lp(a) categories (adjusted Wald test)

Although subjects were unaware of their Lp(a) level, individuals with higher Lp(a) concentrations tended to be more often aware of their dyslipidemia and to receive treatment for it (Fig. 2). Among individuals with dyslipidemia, unawareness was reported in 47.5% of men and 48.4% of women with low Lp(a), compared to 42.3% of men and 38.8% of women with elevated Lp(a) (p for difference 0.180 in men and 0.042 in women).

Among men with dyslipidemia, lipid-lowering medication was received by 38.1% of those with elevated Lp(a) and 31.7% of those with low Lp(a) (*p* = 0.010). Among women, the results were similar in direction, but the difference did not reach statistical significance (29.2% vs. 24.7%, *p* = 0.090). Of those treated, control of dyslipidemia was achieved in 81.4% of men with elevated Lp(a) and 84.1% of men with low Lp(a) (*p* = 0.520). Among treated women, 74.6% of those with elevated Lp(a) and 73.0% of those with low Lp(a) achieved control of dyslipidemia (*p* = 0.740).

## Discussion

The results of this study show that approximately 11% of Finnish adults have Lp(a) levels ≥ 125 nmol/L, a threshold associated with increased cardiovascular risk. Elevated Lp(a) was associated with a higher prevalence of dyslipidemia prior to Lp(a) correction, while no associations were found with other cardiovascular risk factors. Although subjects were unaware of their Lp(a) level, individuals with elevated Lp(a) showed slightly greater awareness and treatment of dyslipidemia. Dyslipidemia control among treated individuals was comparable between those with high and low Lp(a) levels, although overall control rates were not optimal.

It is well known that the distribution of Lp(a) differs between ethnic groups [19]. Previously, results from the Young Finns Study showed that elevated Lp(a) levels were present in fewer than 10% of participants [20]. Consistent with these findings among adolescents, we observed that elevated Lp(a) levels are present in approximately one in ten Finnish adults, which is only about half of the prevalence reported in most other European countries [21]. For example, in the Danish and Polish populations, the proportion of individuals with elevated Lp(a) is approximately 20% [22–24]. However, despite this lower population-level Lp(a) burden, the standardized total death rates from diseases of the circulatory system in 2021 were 211.8 in Denmark and 304.0 in Finland [25].

Although there is now extensive evidence establishing elevated Lp(a) as an independent risk factor for CVD, data on the association between circulating Lp(a) levels and cardiometabolic risk factors in the general population remain limited. Regarding blood pressure, we did not observe an association between Lp(a) and blood pressure, and this aligns with previous, although limited, literature showing no consistent links between Lp(a) concentrations and hypertension prevalence or blood pressure levels [26–28]. Lp(a) has, however, been shown to modify cardiovascular risk among individuals with hypertension. The strongest evidence comes from the community-based, multi-ethnic MESA cohort (*n* = 6,674, median follow-up ≈ 14 years), which demonstrated that hypertension was the primary driver of cardiovascular events, yet elevated Lp(a) amplified this risk: among hypertensive participants, higher Lp(a) levels conferred additional cardiovascular risk compared with lower levels, whereas among non-hypertensive individuals, elevated Lp(a) did not materially increase risk relative to the reference group [26]. Supporting this interpretation, elevated Lp(a) has also been linked with adverse nocturnal and pulse-pressure BP profiles in hypertensive adults [27], suggesting that the interaction between Lp(a) and hypertension extends beyond conventional office BP measurements. Overall, the available evidence supports the interpretation that Lp(a) acts primarily as a cardiovascular risk amplifier in the presence of hypertension, rather than as an independent determinant of hypertension.

The relationship between Lp(a) and diabetes risk is less well established than its role in cardiovascular disease. In our study, elevated Lp(a) concentrations were not associated with abnormal glucose metabolism, consistent with earlier population-based findings reporting no direct or consistent link between Lp(a) and abnormal glucose metabolism [29–32]. Notably, some studies have reported that lower circulating Lp(a) levels are associated with a higher risk of type 2 diabetes, although the temporality and underlying mechanisms remain uncertain [31, 32]. Additional support comes from the ODYSSEY OUTCOMES post-hoc analysis, where low baseline Lp(a) predicted higher diabetes risk and alirocumab-induced reductions in Lp(a) and LDL-C showed opposite associations with diabetes, yet without increasing overall diabetes incidence during PCSK9 inhibition [33, 34]. Together with Mendelian randomization findings [35] linking genetically higher fasting insulin to lower Lp(a) levels, these results suggest that very low Lp(a) may reflect underlying insulin-resistant metabolic states rather than exert a causal effect on glucose dysregulation. Although certain metabolic interventions, such as pioglitazone [36] and bariatric surgery [37, 38], can modestly lower Lp(a), it remains uncertain whether such changes have any meaningful relevance for glucose metabolism. In line with this, and consistent with earlier evidence [39], we found no association between Lp(a) levels and either BMI or waist circumference, further supporting the view that Lp(a) is largely independent of overall or central adiposity. Taken together, current evidence suggests that Lp(a) is not consistently associated with abnormal glucose metabolism; instead, very low Lp(a) may act as a biomarker of insulin resistance rather than a causal driver of diabetes. Ongoing phase 3 trials of potent Lp(a)-lowering therapies will be crucial for determining whether substantial and sustained reductions in Lp(a) influence glucose homeostasis or the risk of new-onset diabetes.

In this study, individuals with elevated Lp(a) appeared to have better awareness and had more often treatment for dyslipidemia, despite being unaware of their Lp(a) levels at the time of examination. This suggests that factors other than elevated Lp(a), most likely prior recognition of dyslipidemia, explain the observed differences. Although the population-based design reduces the likelihood of healthcare-seeking bias, participants with more severe lipid abnormalities may still have been more likely engaged in preventive care. Nevertheless, their cholesterol levels remained equally poor in control, indicating that the contribution of Lp(a) to total and LDL-related cholesterol fractions is only modestly responsive to statin therapy. Importantly, irrespective of Lp(a) levels, one in five men and one in four women using lipid-lowering medication did not achieve LDL-C treatment targets. Together these findings underscore persistent gaps in lipid management at the population level and ensuring identification of individuals with elevated Lp(a). Although several promising Lp(a)-targeted agents, including siRNA therapies, pelacarsen, and the oral inhibitor muvalaplin, are being investigated in clinical trials, their long-term efficacy and safety in cardiovascular prevention still need to be established to ensure clinical implementation [40].

Current cardiovascular prevention guidelines emphasise LDL-C based risk assessment and recommend a one-time Lp(a) measurement to refine risk stratification. Although correcting LDL-C for Lp(a) is not advised — because measured and calculated LDL-C inherently include Lp(a) and the proportion of Lp(a) varies widely — the implications of elevated Lp(a) for dyslipidemia classification remain complex and unresolved. In this context, our population-based findings show that dyslipidemia was more common among individuals with elevated Lp(a), suggesting that conventional LDL-C partly reflects Lp(a)-derived cholesterol. Yet after adjusting LDL-C for Lp(a), the association weakened in men and reversed in women, and elevated Lp(a) was similarly linked to a lower prevalence of high LDL-C after correction. These preliminary results align with earlier literature suggesting that Lp(a)’s contribution to measured LDL-C may meaningfully influence lipid classification and treatment decisions [41]. However, on the other hand, evidence consistently indicates that conventional rather than Lp(a)-corrected LDL-C predicts cardiovascular events, except at very high Lp(a) levels [26, 42]. Taken together, these observations underscore the need for clearer clinical frameworks on how Lp(a) should be incorporated into dyslipidemia assessment and cardiovascular risk prediction and highlight the importance of further robust research to guide optimal risk stratification and management.

The present study provides a comprehensive analysis of Lp(a) and its associations with other cardiovascular risk factors. Key strengths of our study include the use of a large, nationally representative sample, which enhances the generalizability of our findings. Furthermore, the application of standardized and rigorous measurements of cardiovascular risk factors together with the inclusion of key cardiometabolic indicators provides a robust and reliable framework for exploring the complex interplay between these metabolic health outcomes.

Despite its strengths, this study has several limitations. First, due to its cross-sectional design, causal relationships between Lp(a) levels and cardiometabolic risk factors cannot be established. Second, although the study includes a broad range of cardiovascular risk markers, it does not incorporate individual-level risk stratification or personalized lipid targets. This reflects the nature of population-based research, which aims to describe associations at the group level rather than provide individualized risk assessments. Developing personalized risk profiles would require more detailed clinical data and would introduce additional complexity and uncertainty, particularly regarding data completeness and reliability. Third, although the sample is nationally representative and the participation rate was relatively high, especially by international standards, certain subgroups may still be underrepresented, potentially limiting the generalizability of subgroup-specific findings. To mitigate this limitation, we incorporated register-based data, including information from non-participants, and applied inverse probability weighting to correct for non-participation. This approach enhances the generalizability of our findings to the broader population. Finally, it is important to acknowledge the potential for survival bias. Individuals with markedly elevated Lp(a) levels may have experienced early, fatal cardiovascular events and therefore were no longer available to participate in the study. This may not only lead to an underestimation of the association between elevated Lp(a) levels and the overall burden of cardiovascular disease in the population but may also complicate the investigation of these associations.

## Conclusions

This large population-based study of over 5,500 middle-aged adults revealed that elevated Lp(a) levels were present in approximately one in ten men and women, suggesting that elevated Lp(a) concentrations may be less prevalent in Finland compared to many other populations. Elevated Lp(a) was associated with a higher prevalence of dyslipidemia prior to Lp(a) correction, while no associations were found with other cardiovascular risk factors. Although individuals with elevated Lp(a) showed slightly better awareness and treatment of dyslipidemia than those with lower levels, lipid management remained suboptimal across both groups, underscoring the continued importance of effective LDL-C reduction as a primary preventive strategy. From a public health perspective, these findings highlight the need for comprehensive cardiovascular risk assessment, together with sustained efforts to optimize the management of modifiable risk factors. 

## Supplementary Information

Supplementary Material 1.

## Data Availability

Healthy Finland data is not publicly available as it includes confidential information that could compromise the privacy of the participants. The data can be used for research and monitoring of health, wellbeing, functioning, and use of services of the population at THL, and with collaborators based on collaboration agreement; for more information see https://thl.fi/en/research-and-development/research-and-projects/healthy-finland-survey/information-for-researchers. The data available from the THL Biobank cover those who have participated in the health examination and have given consent to biobanking and can be applied in accordance with the Biobank Act and THL Biobank research areas [thl.fi/biobank]. The Finnish Social and Health Data Permit Authority, Findata, may grant permits in accordance with the act on the secondary use of social and health data in Finland [www.findata.fi/en]. Further enquiries can be directed to the email address [tervesuomi@thl.fi].
