# Supplementary material for: Lipoprotein(a) levels in Finnish adults: distribution and associations with other cardiovascular risk factors and their awareness and control
Source: Lipids Health Dis. 2026 Apr 2;25:128. doi: 10.1186/s12944-026-02938-x (PMC13170023; doi:10.1186/s12944-026-02938-x)
Supplement: Supplementary file 1 — Supplementary Material 1. [file 12944_2026_2938_MOESM1_ESM.pdf]

# Lipoprotein(a) levels in Finnish adults:

## Distribution and associations with other cardiovascular risk factors and their awareness and control

Alpo Vuorio, Anniina Ojanen, Tarja Palosaari, Tuija Jääskeläinen, Pekka Jousilahti, Maija Ruuth, Terhi Vihervaara, Mari Savolainen, Lara Lehtoranta, Petri T Kovanen, Annamari Lundqvist

### The study population included 5484 individuals aged 20 or over*

*The data has been collected from Healthy Finland Study (2023).

**11% of the Finnish general adult population have elevated Lp(a) (≥125 nmol/L)**

**11%**

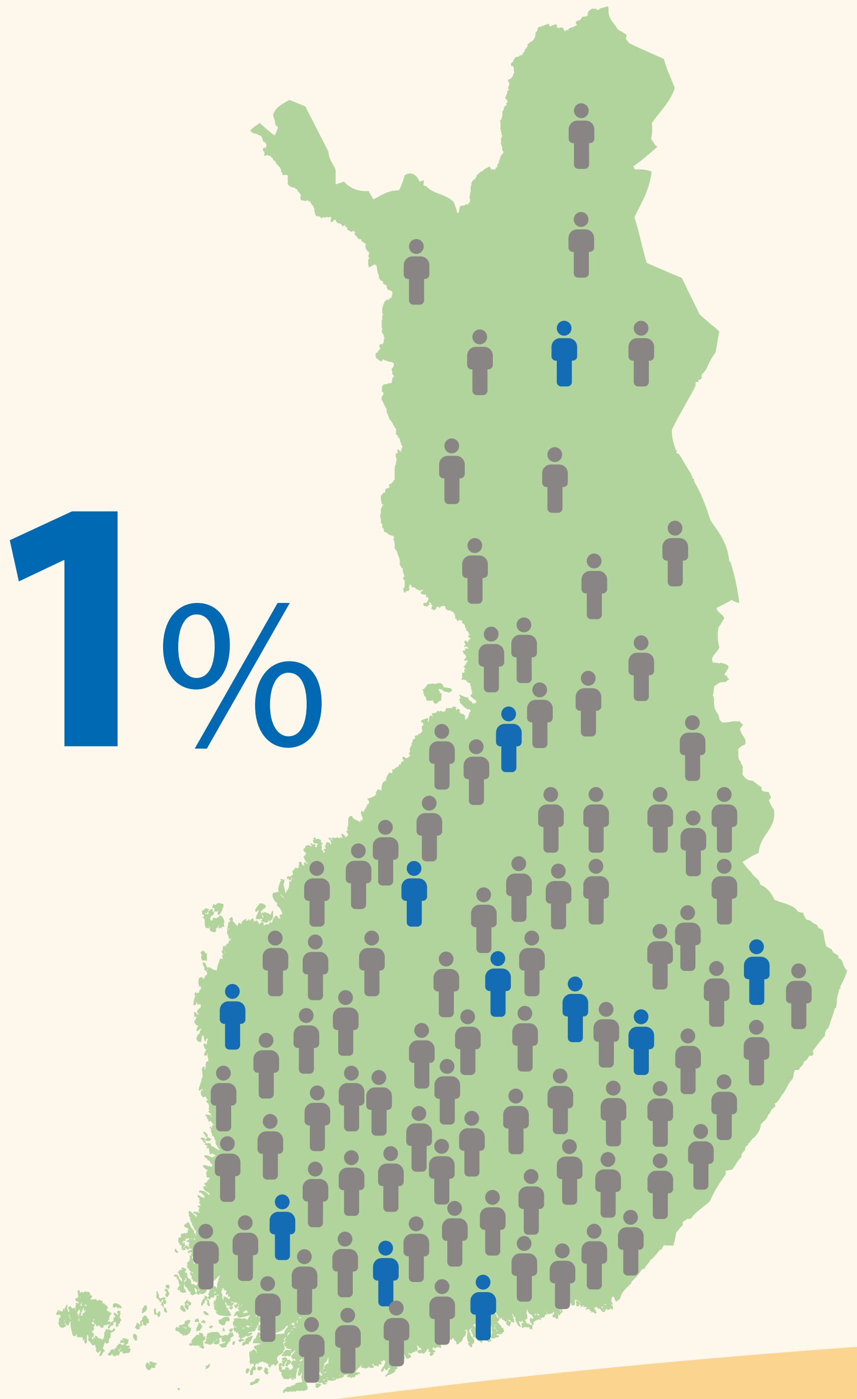

**Elevated Lp(a) was associated with higher prevalence of dyslipidemia prior LDL correction, but not with other CVD risk factors**

| | Men | | | Women | | |
|---|---|---|---|---|---|---|
| | Lp(a) < 125 nmol/L | Lp(a) ≥ 125 nmol/L | | Lp(a) < 125 nmol/L | Lp(a) ≥ 125 nmol/L | |
| **n** | **2 222** | **284** | | **2 657** | **321** | |
| **Cardiometabolc risk factors** | **%** | **%** | **p** | **%** | **%** | **p** |
| Dyslipidemia | 78.4 | 88.1 | * | 73.2 | 79.2 | * |
| Dyslipidemia corrected with Lp(a) | 75.7 | 72.1 | NS | 70.0 | 62.6 | * |
| Abnormal glucose metabolism | 13.8 | 11.7 | NS | 9.7 | 9.2 | NS |
| High blood pressure | 52.5 | 49.4 | NS | 43.6 | 47.4 | NS |
| Obesity | 26.7 | 23.5 | NS | 29.0 | 30.8 | NS |
| Abdominal obesity | 39.3 | 35.7 | NS | 52.7 | 54.4 | NS |
| **Accumulation of cardiometabolic risk factors** | **%** | **%** | **p** | **%** | **%** | **p** |
| At least one | 86.9 | 91.5 | NS | 82.8 | 86.6 | NS |
| At least two | 59.1 | 61.7 | NS | 58.7 | 62.6 | NS |
| 3-4 other risk factors | 30.9 | 25.7 | * | 31.5 | 32.6 | NS |

**Individuals with elevated Lp(a) tended to have better awareness and treatment of dyslipidemia despite not knowing their Lp(a) level**

The prevalence of unawareness, lipid-lowering treatment and control of LDL-cholesterol (<2.6 mmol/l) according to Lp(a) level.

| | Men: Lp(a) < 125 nmol/L | Men: Lp(a) ≥ 125 nmol/L | Women: Lp(a) < 125 nmol/L | Women: Lp(a) ≥ 125 nmol/L |
|---|---|---|---|---|
| Unaware | 47.5% | 42.3% | 48.4% | 38.8%† |
| Treatment | 31.7% | 38.1%† | 24.7% | 29.2% |
| Controlled lipids | 84.1% | 81.4% | 73.0% | 74.6% |

● Lp(a) < 125 nmol/L ● Lp(a) ≥ 125 nmol/L

†p-value <0.05
Among those on lipid-lowering medication
CVD, cardiovascular disease

**Although elevated Lp(a) does not associate with worse cardiovascular risk factor profile, overall risk factor levels were high. These findings underscore the importance of ensuring better awareness and optimal management of modifiable risk factors to reduce the overall burden of CVD.**
